# Bio-behavioral Interventions for Cardiovascular Diseases Rehabilitation: A Systematic Review on Heart Rate Variability Biofeedback and Nutrition

**DOI:** 10.1007/s10484-025-09742-w

**Published:** 2025-10-21

**Authors:** Valeria Carola, Valeria Gigli, Filippo Cellucci, Marco Coli, Sofia Nicolais, Caterina Piras, Giovanni Melina, Cristina Ottaviani, Giampaolo Nicolais

**Affiliations:** 1 Department of Dynamic and Clinical Psychology, and Health Studies, Sapienza University of Rome, Via degli Apuli, 1, 00185 Rome, Italy; 2 Department of Psychology, Sapienza University of Rome, Via dei Marsi, 78, 00185 Rome, Italy; 3 Department of Clinical and Molecular Medicine, Sapienza University of Rome, Via di Giorgio Nicola Papanicolau, 00189 Rome, Italy; 4 Department of Experimental Medicine, Sapienza University of Rome, Viale Regina Elena, 324, 00161 Rome, Italy; 5 Department of Human Sciences and Promotion of Quality of Life, San Raffaele Telematic University, Via di Val Cannuta, 247, 00166 Rome, Italy; 6 IRCCS Santa Lucia Foundation, Via Ardeatina, 306, 00179 Rome, Italy

**Keywords:** Cardiovascular disease, Rehabilitation, Prevention, Heart rate variability biofeedback, Nutrition

## Abstract

Cardiovascular diseases (CVD) are leading causes of premature mortality, disability, and reduced quality of life worldwide. While extensive literature has addressed cardiovascular risk and treatment, the role of noninvasive interventions in rehabilitation and secondary prevention remains less well defined. Among these, heart rate variability biofeedback (HRVBF) and cardioprotective dietary approaches have shown preventive benefits, yet their effectiveness in patients with established CVD or those undergoing cardiac rehabilitation is not fully established. This review systematically evaluates the evidence for HRVBF and heart-healthy dietary patterns in relapse prevention and rehabilitation, with particular attention to hospital-based contexts. Following PRISMA guidelines, systematic searches were conducted in Scopus and PubMed. Results suggest that HRVBF may enhance autonomic regulation, alleviate psychological distress, and shorten hospital stays. Dietary interventions, particularly those emphasizing omega-3 polyunsaturated fatty acids and Mediterranean-style patterns, demonstrate potential to reduce inflammation and modulate cardiovascular risk markers. Nevertheless, evidence supporting their integration into postoperative rehabilitation or treatment of established CVD remains limited. Heterogeneity in study designs and lack of standardization further constrain generalizability. Overall, the findings underscore the promise of early, noninvasive, and integrative rehabilitation strategies, highlighting the need for structured protocols in clinical settings, particularly during the immediate postoperative phase or after acute cardiac events.

**Graphical Abstract:**

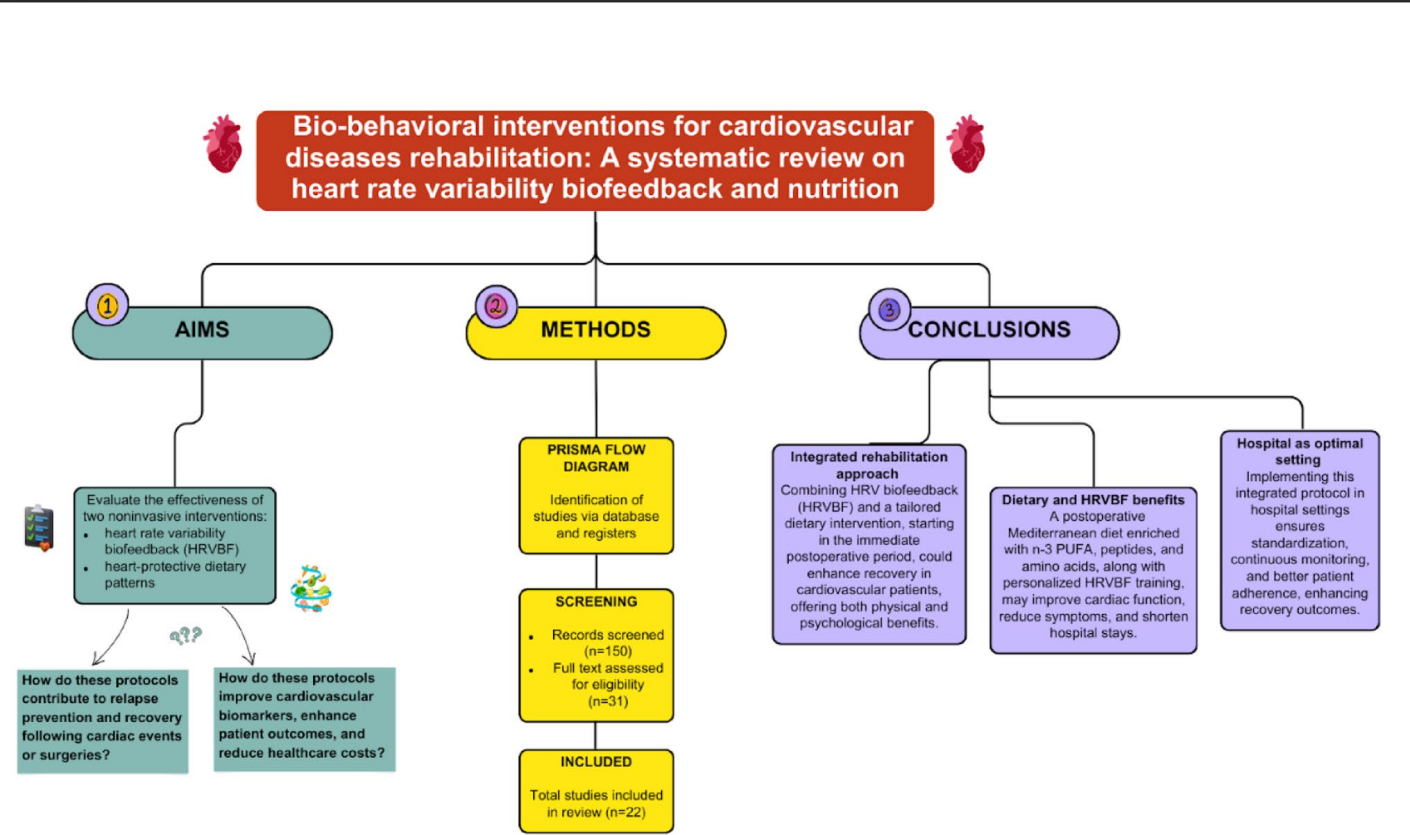

## Introduction

### Risk Factors, Prevention Strategies, and Emerging Approaches for Cardiovascular Diseases

Cardiovascular diseases (CVD) remain the leading cause of premature mortality worldwide (Teo & Rafiq, 2021). Epidemiological evidence highlights their profound impact on global health, with CVD responsible for approximately 7.4 million and 6.7 million deaths annually, in addition to substantial morbidity, disability, and diminished quality of life (Hajar, 2016; Roth et al., 2020). These conditions are multifactorial, arising from both non-modifiable risk factors (e.g., genetics, age, sex, ethnicity) and modifiable behavioral and lifestyle determinants (Hajar, 2016).

Among modifiable factors, lifestyle behaviors play a central role in CVD pathogenesis. According to the World Health Organization (WHO), nearly three-quarters of cardiovascular mortality could be prevented through healthy lifestyle choices, including regular physical activity (Elkind, 2024). Tobacco use, physical inactivity, and unhealthy diets are strongly linked to systemic inflammation and chronic disease progression (Arafa et al., 2021).

While primary prevention—reducing risk before disease onset—has been extensively studied (Stewart et al., 2017), secondary and tertiary prevention strategies remain comparatively underexplored. Secondary prevention targets early detection and recurrence reduction through pharmacological management and patient education (Karunathilake & Ganegoda, 2018). Tertiary prevention seeks to limit complications and disability in individuals already diagnosed with CVD, often involving rehabilitation programs and long-term management (Prasad, 2021). Identifying stage-specific, effective strategies is therefore a public health priority (Labarthe, 2011), both to improve outcomes and reduce healthcare costs (Gaziano, 2005).

In recent years, increasing attention has been devoted to physiological indices that may inform prevention, diagnosis, and rehabilitation of CVD. Among these, heart rate variability (HRV) has emerged as a promising biomarker of cardiovascular risk, disease progression, and treatment response (Fang et al., 2020). HRV reflects beat-to-beat fluctuations in heart rate, indexing the dynamic balance between sympathetic and parasympathetic activity (Electrophysiology, 1996; Laborde et al., 2017). Preclinical studies support its association with vagal nerve activity (Ter Horst & Postema, 1997). Reduced HRV has consistently been linked to autonomic imbalance (Thayer et al., 2010; Tsuji et al., 1996) and adverse outcomes, including coronary heart disease, sudden cardiac death (Dekker et al., 1997; Hon, 1963), and post-myocardial infarction (MI) mortality (Carney et al., 2005).

The Neurovisceral Integration Model (Thayer & Lane, 2000) further positions HRV as a proxy of prefrontal cortex functioning, emotion regulation, and adaptability (e.g., Quintana et al., 2012). HRV indices are derived from both time- and frequency-domains analyses. Time-domain measures such as SDNN (standard deviation of normal-to-normal intervals) reflect overall variability, while RMSSD (root mean square of successive differences between normal heartbeats) primarily reflects parasympathetic modulation. Frequency-domain indices assess power across specific frequency bands: very-low (VLF-HRV) and low-frequency (LF-HRV) components relate to baroreflex function, while high-frequency (HF-HRV) power reflects vagal modulation of heart rate (Hadase et al., 2004; Shaffer & Ginsberg, 2017). The LF/HF ratio remains debated as a marker of autonomic balance (Billman, 2013).

In parallel, nutrition plays a critical role in both prevention and progression of CVD. Diets rich in high in ultra-processed foods—high in refined carbohydrates, added sugars, trans fats, and sodium—are associated with a 35%, increased risk of CVD (Juul et al., 2021; Timmis et al., 2022), largely mediated by pro-inflammatory pathways (Silveira et al., 2018). By contrast, diet emphasizing fruits, vegetables, whole grains, legumes and lean proteins have demonstrated anti-inflammatory effects and lower mortality (Diab et al., 2023). The Mediterranean diet, in particular, improves lipid profiles, modulates pro-atherogenic genes, alters gut microbiota composition and reduces systemic inflammation (Diab et al., 2023). Nutrigenomic studies further suggest that adherence to cardioprotective dietary patterns may attenuate genetic risk for adverse cardiometabolic phenotypes (Richardson et al., 2022).

Despite well-established roles of HRV (Hillebrand et al., 2013) and nutrition in cardiovascular health, their integration into secondary and tertiary prevention protocols, particularly in patients with established CVD or post-surgical recovery, remains limited. Addressing this gap is crucial to reducing invasive procedures such as angioplasty and coronary artery bypass grafting (CABG), and to preserving quality of life.

### Treatment and Rehabilitation Opportunities: Heart Rate Variability Biofeedback and Cardioprotective Nutrition

CVD treatment strategies vary by condition and severity, ranging from lifestyle changes to pharmacological risk management (Raimondo et al., 2022). In many cases, cardiac surgery, including CABG, aortic valve replacement, and mitral valve repair, is necessary (Allam & Akowuah, 2024). These interventions have high success rates (96–97%, NICE) and significantly improve survival (Allam & Akowuah, 2024; Kostis, 1997; World Health Organization, 2017).

Post-surgery rehabilitation is critical to recovery, relapse prevention, and long-term well-being. Standard programs combine exercise training, pharmacotherapy, and patient education. While structured physical activity is highly effective (Anderson et al., 2016), its feasibility in the immediate postoperative phase is often limited by pain, fatigue, reduced mobility, or sternal instability (Abreu et al., 2019; Sabbahi et al., 2022). This underscores the need for safe, low-burden interventions suitable for early recovery.

Psychological distress further complicates recovery: up to 40% of cardiac patients experience depression, anxiety, or stress after acute events or surgery (Celano et al., 2016; Dickens, 2015; Silverman et al., 2019; Steptoe & Kivimäki, 2013). Psychosocial interventions—including counseling and stress management—have shown efficacy in improving cardiovascular outcomes and adherence to rehabilitation (Arnett et al., 2019; Richards et al., 2018; Klainin-Yobas et al., 2016; Nijjar et al., 2014). Mind-body interventions such as mindfulness, yoga, and biofeedback, have also demonstrated efficacy in improving HRV, emotional regulation, and pulmonary function (Bigger et al., 1992; Casolo et al., 1989; Kaur et al., 2014; Mehregan-Far et al., 2024). Nutritional interventions also play a key role in secondary prevention, though their implementation in postoperative care remains inconsistent (Hotta et al., 2021).

We focus on two promising, non-invasive interventions—HRVBF and cardioprotective diets—which share three core strenghts: (1) feasibility during early recovery, (2) non-invasive and cost-effective implementation, and (3) targeting of shared physiological mechanisms, particularly autonomic regulation and inflammation.

HRVBF employs paced breathing (~ 6 breaths/minute) to enhance vagal tone, improve HRV, and optimize respiratory sinus arrhythmia (Christine, 2008; Lehrer & Gevirtz, 2014; Moravec & McKee, 2011; Yu et al., 2018). Inhalation transiently suppresses vagal input, increasing heart rate, while exhalation restores vagal activity, lowering it. This resonance process fosters autonomic balance, reduces distress, and may shorten hospital stays (Del Pozo et al., 2004; Moss & Shaffer, 2017).

Similarly, cardioprotective dietary patterns, particularly the Mediterranean diet, offer anti-inflammatory, endothelial-protective benefits and reduces recurrent events in individuals with CVD (Diab et al., 2023). Contextually, this kind of intervention could be easily implemented during the early postoperative period. Yet, systematic implementation in rehabilitation programs remains limited, hindered by protocol variability, poor standardization, and adherence challenges (Kocanda et al., 2023; Lacroix et al., 2017).

While these interventions differ in modality, they converge mechanistically by targeting the autonomic nervous system and inflammation. Dysregulation of the autonomic nervous system has consistently been associated with heightened inflammatory responses, impaired vascular integrity, and poorer cardiovascular outcomes (Thayer & Sternberg, 2006). By addressing these pathways, both HRVBF and anti-inflammatory dietary patterns have the potential to create a favorable physiological milieu for recovery, simultaneously reducing psychophysiological stress, supporting vascular function, and modulating immune activity. This convergence is particularly relevant given the multifactorial nature of CVD recovery, which rarely involves only physiological impairment but often includes psychological and behavioral dysregulation as well.

Most available studies have concentrated on the preventive role of these interventions in long-term risk reduction, whereas only a limited number have investigated their application during the immediate post-surgical rehabilitation phase (Bolin et al., 2022; Mozaffarian, 2016). Yet, their non-invasive profile, adaptability and feasibility in clinical contexts make them especially well-suited for hospital-based settings and for use during the early recovery period, when more intensive interventions are often impractical.

Designing rehabilitation protocols for this critical phase requires careful consideration of patients’ clinical status. After major cardiac procedures, many individuals present with significant mobility restrictions due to frailty, fatigue, or surgical complications such as sternal instability. Under such conditions, high-intensity exercise-based approaches—although shown to be highly effective—may not be feasible or safe (Abreu et al., 2019; Sabbahi et al., 2022; Williams et al., 2007). In contrast, HRVBF and nutritional strategies offer low-burden, flexible approaches that can be initiated early and tailored to each patient’s functional capacity.

Nonetheless, the translation of these approaches into standard rehabilitation practice remains limited. Research in this area is fragmented, with substantial heterogeneity in study design, timing of interventions, and outcome measures. The absence of standardized protocols and clear guidelines restricts their integration into evidence-based clinical programs. Addressing these limitations is not only crucial for advancing scientific understanding but also for informing practical, real-world clinical decisions. Incorporating HRVBF and cardioprotective dietary interventions into rehabilitation pathways could provide cost-effective and scalable strategies to reduce re-hospitalization, improve patients’ quality of life, and ultimately decrease long-term mortality.

This systematic review consolidates and critically evaluates the evidence on HRVBF and cardioprotective dietary interventions in secondary and tertiary CVD prevention, with a special focus on their application in early postoperative and hospital-based rehabilitation. By highlighting current evidence gaps and mechanistic insights, we aim to inform the development of integrative, sustainable rehabilitation strategies.

## Methods

### Search Strategy

A systematic search of PubMed and Scopus were screened (up to July 24, 2024; updated May 1, 2025) in accordance with PRISMA (Preferred Reporting Items for Systematic Reviews and Meta-Analyses) guidelines. The review focused on bio-behavioral rehabilitation methods involving HRV modulation (HRVBF or deep breathing techniques) and nutritional interventions in patients with established CVD or post-surgical recovery. An initial combined search including both HRVBF and nutrition terms was piloted but yielded very limited and no eligible results. Given the distinct indexing of the two literatures, we therefore conducted separate searches for each domain, complemented by manual cross-checking and citation tracking to minimize the risk of missing integrated approaches.

Separate searches were then run for HRVBF and dietary interventions in prevention and rehabilitation contexts using predefined Boolean strings. For studies on HRV modulation in prevention strategies, a search was conducted on July 24, 2024, using the following search string: (“cardiovascular surgery*” OR “cardiac surgery” OR “coronary artery bypass graft surgery” OR “valve replacement” OR “valve repair”) AND (“heart rate variability” OR “autonomic nervous system” OR “sympathetic nervous system” OR “respirat* biofeedback” OR “resonance frequency biofeedback” OR “heart rate variability biofeedback”) AND “prevention*”.

For HRV modulation studies in rehabilitation, a second search on July 24, 2024, used the following keywords included in the title or abstract: (“cardiovascular surgery*” OR “cardiac surgery” OR “coronary artery bypass graft surgery” OR “valve replacement” OR “valve repair”) AND (“heart rate variability” OR “autonomic nervous system” OR “sympathetic nervous system” OR “respirat* biofeedback” OR “resonance frequency biofeedback” OR “heart rate variability biofeedback”) AND “rehabilitation*”.

To explore dietary interventions in prevention strategies, the search on July 24, 2024, used this string: (“cardiovascular surgery*” OR “cardiac surgery” OR “coronary artery bypass graft surgery” OR “valve replacement” OR “valve repair”) AND (“diet” OR “nutritional management”) AND “prevention*”.

Lastly, for nutrition-related rehabilitation studies, the search conducted on July 24, 2024, used the following string: (“cardiovascular surgery*” OR “cardiac surgery” OR “coronary artery bypass graft surgery” OR “valve replacement” OR “valve repair”) AND (“diet” OR “nutritional management”) AND “rehabilitation*”.

In order to ensure inclusion of the most recent and relevant studies, an updated bibliographic search was conducted on May 1, 2025, using the same four search strings in PubMed and Scopus. The results of this updated search are reported separately, both within the main text and in the PRISMA flow diagram.

### Eligibility Criteria

Studies were included if they:

- involved adults (older than 18 years) with established cardiac conditions (e.g., coronary artery disease, heart failure, MI) or experiencing other acute cardiac events or patients undergoing cardiac surgery (CABG, valve replacement/repair). These surgery procedures were prioritized due to their prevalence in clinical practice and their substantial impact on cardiovascular recovery and rehabilitation;
- evaluated HRVBF or respiratory techniques for autonomic regulation, or dietary modifications/supplementation;
- compared these interventions with standard care, alternative rehabilitation strategies, or control conditions;
- used cross-sectional, randomized control trial, or pilot designs;
- were published in English, peer-reviewed journals.

### Exclusion Criteria

The following types of studies were excluded from the review:

- Reviews, meta-analyses, single case reports, correlational studies;
- Studies on primary prevention only, with no focus on established CVD or rehabilitation;
- Pharmacological or supplement-only interventions;
- Studies focusing solely on psychological or atrial fibrillation outcomes;
- Protocol papers without empirical data;
- Lifestyle programs lacking HRVBF or dietary components.

### Study Selection and Data Extraction

Studies selection was carried out by five authors (MC, VG, SN, FC, CP), who independently screened titles and abstracts before performing full-text reviews. Duplicate records were identified and removed manually. To minimize selection bias, at least three authors (VG, FC, CP) cross-checked the database for errors or inconsistencies during the selection process.

For each eligible study, data were extracted using a structured form, including: study design, sample size, participant characteristics (age, sex), intervention type and timing (HRVBF or dietary), control/comparison conditions (where present), primary outcomes and main findings. Outcomes were not pre-specified a priori; instead, those reported in the studies were collected. These included psychological outcomes (e.g., anxiety, depression), psychophysiological measures (e.g., HRV, RSA), and clinical endpoints (e.g., body mass index, lipid profile, postoperative atrial fibrillation (POAF). Only a minority of studies reported long-term outcomes such as readmission or mortality. Notably, one trial (Yu et al., 2018) documented all-cause readmissions and emergency visits at 1-year follow-up, with no deaths reported. Where available, additional information such as inclusion/exclusion criteria, intervention setting, and follow-up duration was also extracted. Data extraction was performed independently by multiple authors, with discrepancies resolved by discussion.

## Results

The initial search (July 24, 2024) identified 160 records (38 from PubMed, 122 from Scopus), plus 12 additional studies identified through manual reference screening. After removal of 10 duplicates, 162 records were screened. Based on predefined eligibility criteria, 76 were excluded during title/abstract screening. A further 64 were excluded because they were reviews, meta-analyses, conference abstracts, study protocols, or lacked sufficient methodological detail or results. Additional exclusions involved pediatric/adolescent populations, studies not employing the target interventions, or those without relevant outcomes. This process yielded 22 eligible studies, which formed the initial review database (see Tables 1, 2).

To ensure coverage of recent literature, the search was updated on May 1, 2025, applying the same four search strings across PubMed and Scopus for studies published after July 24, 2024. This yielded 19 new records. After removal of one duplicate, 18 records were screened. Of these, 17 were excluded for not meeting inclusion criteria (e.g., wrong intervention, irrelevant outcome). One additional study satisfied all criteria and was included, bringing the final dataset to 23 studies.

The complete study selection process for both the initial and updated searches is illustrated in the PRISMA flowchart. All included studies are summarized in Tables 1 and 2. Collectively, the 23 studies involved approximately 2774 participants, with sample sizes ranging from 10 to 1516. Reported samples were predominantly males (54–100%) with mean ages between 54 and 72 years. However, many studies did not provide detailed demographic characteristics, limiting the ability to describe the pooled sample comprehensively. Most studies recruited adults undergoing cardiac surgery, particularly CABG and valve replacement. Typical inclusion criteria involved a diagnosis of coronary artery disease and recent cardiac surgery, while exclusion criteria frequently ruled out psychiatric disorders, cognitive impairment, or severe comorbidities.

**Fig. 1:**
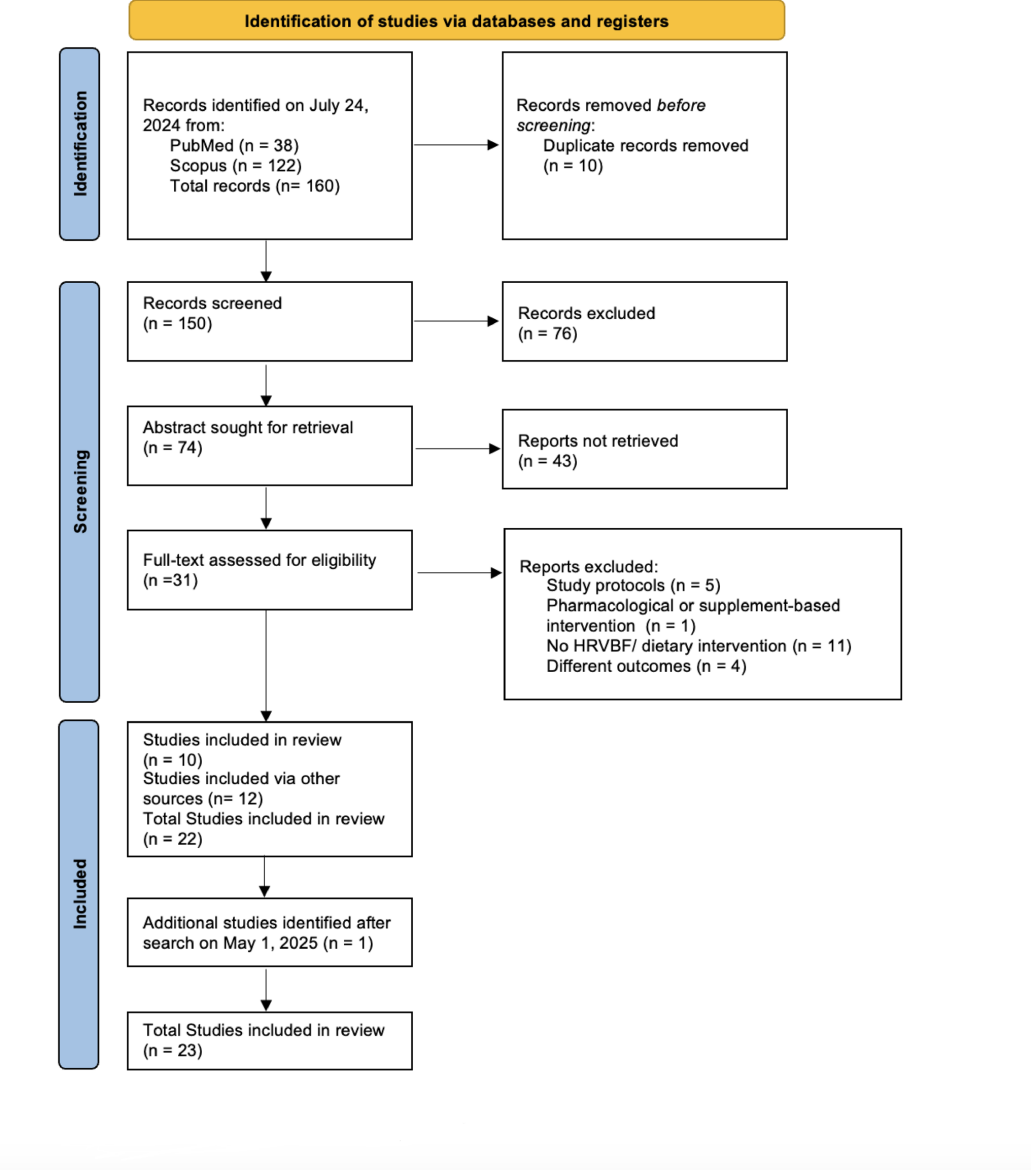
PRISMA flow diagram showing study selection

The detailed findings of the included studies are presented in the following sections (Fig. 1).

### Heart Rate Variability Biofeedback in Cardiovascular Disease Management and Rehabilitation

A large body of research on secondary and tertiary prevention in coronary patients has shown that optimized cardioprotective drug therapy alone does sufficiently reduce cardiovascular risk factors (Bacquer et al., 2022; Moerschel et al., 2022). Cardiac rehabilitation—most notably exercise training—after an acute event or surgical procedure remains a cornerstone strategy for restoring cardiac function and improving overall performance (Shilton et al., 2024). Within this context, interventions that enhance parasympathetic activity through vagal stimulation and HRV training are increasingly recognized for their potential to improve prognosis and reduce recurrent events. This review therefore examined the role of HRVBF protocols in CVD management and rehabilitation, identifying 12 eligible studies (Adams et al., 2009; Amjadian et al., 2020; Bahrami Ehsan et al., 2019; Climov et al., 2014; Grishin et al., 2015; Limmer et al., 2022; Lin et al., 2015; Moravec & McKee, 2013; Nolan et al., 2005; Swanson et al., 2009; Valdés, 2018; Yu et al., 2018). Most studies assessed long-term outcomes of HRVBF in patients after MI, coronary artery disease (Lin et al., 2015; Nolan et al., 2005; Yu et al., 2018) and chronic heart failure (Moravec & McKee, 2011). Others examined its application after CABG following either pre-existing disease or acute events (Amjadian et al., 2020; Bahrami Ehsan et al., 2019; Climov et al., 2014; Grishin et al., 2015). For example, Lin et al. (2015) conducted a randomized controlled trial in coronary artery disease patients, comparing HRVBF with standard medical care. Their protocol, adapted from Del Pozo and colleagues, Lehrer and Gevirtz, and Swanson and colleagues demonstrated that a 6-week HRVBF intervention significantly increased SDNN and LF-HRV relative to baseline, unlike in controls (Del Pozo et al., 2004; Lehrer & Gevirtz, 2014; Swanson et al., 2009) Similarly, Yu and colleagues found that HRVBF improved short-term HRV indices and was uniquely associated with reduced hospital readmissions at one-year follow-up (Yu et al., 2018). Limmer and colleagues extended this evidence in MI outpatients, reporting that HF-HRV increased and SDNN improved significantly in the HRVBF group, whereas controls showed either no change or decline (Limmer et al., 2022).

Evidence also supports HRVBF following cardiac procedures such as CABG and percutaneous transluminal angioplasty. Studies by Amjadian et al. (2020) and Bahrami Ehsan et al. (2019) reported increases in HRV values post-HRVBF, while Grishin et al. (2015) demonstrated feasibility and noted shorter postoperative hospital stays in CABG patients. By contrast, Adams et al. (2009) found no significant differences between usual rehabilitation and rehabilitation plus controlled breathing, although the high proportion of patients on heart rate–lowering medications (77%) may have obscured treatment effects.

Collectively, these studies suggest that HRVBF enhances parasympathetic modulation, often outperforming standard or adjunctive rehabilitation methods such as exercise, across both chronic and post-acute CVD populations. Nonetheless, an important limitation is that most studies emphasize long-term outcomes, with limited attention to the early postoperative phase. Yet this phase is particularly critical: unlike exercise—which remains the most effective strategy for long-term rehabilitation (Lavie et al., 2015)—HRVBF can be delivered safely even when mobility is restricted by frailty, sternal fractures, or surgical complications. By supporting autonomic recovery during periods of bed rest, HRVBF may reduce the time to initiation of physical rehabilitation and improve overall prognosis.

Additional rationale for HRVBF stems from the established role of respiratory rehabilitation in early recovery, which improves pulmonary function, chest mobility, and reduces postoperative complications (Zanini et al., 2019). It is conceivable that HRVBF, which integrates respiratory modulation, could offer similar early-phase benefits. However, current evidence is insufficient, and robust randomized controlled trials are needed to clarify the short-term efficacy of HRVBF when introduced immediately after surgery.

In summary, HRVBF appears to be a promising adjunct for enhancing autonomic regulation and parasympathetic activity in CVD rehabilitation. Evidence suggests benefits across key HRV parameters (e.g., SDNN, LF-HRV, HF-HRV), with additional reductions in hospital readmissions and length of stay in select populations. While feasibility has been demonstrated, especially after CABG and MI, the lack of studies on early postoperative application underscores an important gap. Addressing this through high-quality trials will be essential to determine HRVBF’s role as a secondary or tertiary prevention strategy in immediate post-surgical rehabilitation (Table 1).

**Table 1:** Studies investigating the efficacy of heart rate variability biofeedback (HRVBF) in cardiovascular disease (CVD) management and rehabilitation

Author (year)	Design	Sample	Pathology/population	Prevention type	Training protocol details; timing	Data analyzed	Results
Nolan et al. (2005)	Randomized controlled trial	46 (Control *n* = 23; HRVBF *n* = 23)	CAD	Secondary	HRVBF 90 min for 5 sessions; After hospital discharge, not specified	Depression, Psychological stress, logHF	Depression ↓, Psychological stress ↓, logHF ↑
Swanson et al. (2009)	Randomized controlled trial	29 (Control *n* = 15; HRVBF *n* = 14)	Heart failure	Secondary	HRVBF 5 min a day for 18 months; <6 weeks post ACS or MI	SDNN, Exercise tolerance, Quality of life	Exercise tolerance ↑
Adams et al. (2009)	Randomized controlled trial	44 (Control *n* = 22; HRVBF *n* = 22)	MI/CABG	Tertiary	HRVBF 10 min twice a day for 8 weeks; 1–8 weeks post CABG	SDNN, Respiratory arrhythmia	SDNN =
Moravec &. McKee (2013)	Pilot study	HRVBF *n* = 20	Heart failure	Secondary	HRVBF 5 min for 6 sessions; Not specified	BR, SDNN	BR ↓, SDNN ↑
Grishin et al. (2015)	Randomized controlled trial	68 (Control *n* = 48; HRVBF *n* = 20)	CABG	Tertiary	Respiratory BF 30 min for 5 sessions; During hospital stay	Postoperative hospital stays, BR	Postoperative hospital stay ↓, BR ↓
Lin et al. (2015)	Randomized controlled trial	127 (Control *n* = 67; HRVBF *n* = 60)	CAD	Secondary	HRVBF 60 min per week for 6 weeks; Not specified	SDNN, LF, log LF, Hostility, BP	SDNN ↑, LF ↑, log LF ↑
Limmer et al. (2022)	Randomized controlled trial	46 (Control *n* = 23; HRVBF *n* = 23)	MI	Secondary	HRVBF 3 × 5 min a day for 12 months; After hospital discharge, not specified	SDNN, BP, HF, LF, HR	SDNN ↑, BP ↓, HR ↓
Valdes et al. (2018)	Randomized controlled trial	20 (Control *n* = 10; HRVBF *n* = 10)	MI	Secondary	HRVBF 5 min/1 session; During hospital stay after MI	SDNN	SDNN ↑
Yu et al. (2018)	Randomized controlled trial	134 (Control *n* = 59; HRVBF *n* = 75)	CAD	Secondary	HRVBF 60 min per week for 1 year; Not specified	Cardiac events, Mortality, Hostility, Depression, SDNN, LF, HF, LF/HF	Cardiac events ↓, Depression ↓, Hostility ↓, SDNN ↑
Climov et al. (2014)	Randomized controlled trial	31 (Control *n* = 15; HRVBF *n* = 16)	CABG/PCI	Tertiary	HRVBF 10 min twice a week for 5 weeks; >2 weeks post ACS; >4 weeks post CABG	Depression, Type D personality, SDNN, BP	SDNN ↑
Bahrami Ehsan et al. (2019)	Randomized controlled trial	40 (Control *n* = 20; HRVBF *n* = 20)	CABG	Tertiary	HRVBF 120 min per week for 8 weeks; After rehab, not specified	Resonance frequency, Stress, Anxiety, Depression	Resonance frequency ↑, Stress ↓, Anxiety ↓, Depression ↓
Amjadian et al. (2020)	Randomized controlled trial	40 (Control *n* = 20; HRVBF *n* = 20)	CABG	Tertiary	HRVBF 120 min per week for 8 weeks; After hospital discharge, not specified	Psycho-physiologic coherence, Anxiety, Depression	Psychophysiological coordination ↑, Anxiety ↓, Depression ↓

*ACS* acute coronary syndrome, *HRVBF* heart rate variability biofeedback, *CAD* Coronary artery disease, *logHF* log-transformed values of high frequency, *MI* myocardial infarction, *SDNN* standard deviation of normal-to-normal intervals, *BR* breathing rate, *CABG* coronary artery bypass grafting, *LF* low frequency, *logLF* log-transformed values of low frequency, *HF* high frequency, *BP* blood pressure, *HR* heart rate, *PCI* Percutaneous coronary intervention, ↓ significant decrease, ↑ significant increase, = no significant variation

### Nutrition in Cardiovascular Disease Management and Rehabilitation

A substantial body of evidence links CVD with obesity, malnutrition, and macro- and micronutrient deficiencies, as well as with the potential benefits of nutritional interventions for both prevention and therapy. Despite this, nutrition has not been systematically integrated into cardiac rehabilitation protocols, and research in this field remains relatively underexplored.

For this reason, the present review examined the role of nutritional interventions in CVD management and rehabilitation, identifying 11 eligible studies (Christensen et al., 1996; Dantas et al., 2002; Feguri et al., 2017; Grundmann et al., 2018; Hodis, 1995; King et al., 2000; Mozaffarian et al., 2008; Mozaffarian et al., 2012; Osterholt et al., 2022; Shingu et al., 2021; Ogawa et al., 2025).

Although nutritional disorders are highly prevalent in heart failure, the available evidence is inconsistent, and major guidelines remain limited in scope (Driggin et al., 2022). Since food composition influences the cardiac autonomic nervous system, it is plausible that diet may modulate HRV. Indeed, vitamin intake has been shown to affect HRV (Young & Benton, 2018). A recent review found associations between HRV and vitamins B12, C, D, and E, as well as with magnesium, iron, zinc, coenzyme Q10 and multivitamin-mineral supplementation (Lopresti, 2020; Strüven et al., 2021a). These findings suggest that diet alone can exert favorable effects on sympatho-vagal balance.

Specific interventions also show promise. Supplementation with vitamins E and C, combined with a cholesterol-lowering diet and colestipol-niacin, may slow coronary lesion progression (Hodis, 1995). Likewise, the combination of dietary change and exercise is important for improving functional capacity in rehabilitation patients (King et al., 2000).

A recent systematic review highlighted the scarcity of high-quality trials on nutrition in cardiac rehabilitation (Kocanda et al., 2021). Nevertheless, available evidence points to positive effects of modifying dietary intake, including increased fruit and vegetable consumption, higher fiber intake, and improvements in scores such as the Dietary Risk Assessment. Benefits were also noted for energy, dairy, carotene, vitamin C, and sodium intake. The review concluded, however, that more targeted research is needed (Kocanda et al., 2021).

Metabolic and nutritional status have been linked to CVD development. At the cellular level, myocardial efficiency is influenced by metabolic condition, overload, and ischemia. In patients with ischemic coronary disease and heart failure, low oxygen availability limits reliance on free fatty acids, increasing glucose utilization. However, excessive intracellular glucose levels are also detrimental (Fukumoto, 2021).

In terms of specific nutrients, patients with acute coronary syndrome frequently present with reduced levels of eicosapentaenoic acid and docosahexaenoic acid, particularly men, independent of age (Fukumoto, 2021). Supplementation with n-3 polyunsaturated fatty acids (PUFA) appears to support secondary prevention in acute coronary syndrome, although evidence remains limited (Fukumoto, 2021).

Some studies have investigated preoperative nutritional interventions. Feguri and colleagues demonstrated the efficacy of limited preoperative fasting combined with carbohydrate loading and intraoperative infusion of n-3 PUFA, a strategy rarely applied in cardiovascular surgery (Feguri et al., 2017). Brief fasting followed by carbohydrate intake two hours before anesthesia improved recovery from CABG procedures and lowered perioperative vasoactive drug requirements. Infusion of n-3 PUFA reduced POAF and shortened hospital stay. Preoperative short-term calorie restriction may also reduce the risk of acute kidney injury after cardiac surgery (Grundmann et al., 2018). More recently, Ogawa and colleagues reported that preoperative supplementation with beta-hydroxy-beta-methylbutyrate, combined with l-glutamine and l-arginine for at least two weeks before cardiac surgery, resulted in greater improvements in six-minute walking distance, muscle strength, and performance, both before and after surgery, and a shorter hospital stay, though no significant differences in muscle mass were observed (Ogawa et al., 2025). By contrast, preoperative short-term restriction of sulfur-containing amino acids did not yield significant benefits in CABG patients (Osterholt et al., 2022). Shingu and colleagues further showed that three oral doses of 1 g L-carnitine (from two days pre-surgery to postoperative day 7) reduced POAF (Shingu et al., 2021).

These findings underscore the potential of nutritional interventions, though more research is needed to achieve consistent, clinically applicable outcomes. For example, while a weight-maintenance low-fat diet improved exercise duration and functional capacity in rehabilitation (King et al., 2000), long-term benefits of a low-fat, low-sugar diet for 180 days post-discharge after CABG were not observed (Dantas et al., 2002). These findings suggest that dietary interventions may be particularly effective in the short-term perioperative period, where compliance is easier to achieve in a structured medical environment. Nonetheless, long-term nutritional strategies remain essential for sustained benefits. Certain biomarkers have been identified as useful indicators in this context (Reginato et al., 2020). HRV modulation is one possible mechanism underlying the beneficial effects of diet on the cardiovascular system, and several dietary factors have been shown to influence HRV both acutely and over time (Young & Benton, 2018).

A broad literature supports the role of n-3 PUFA and B-class vitamins in increasing HRV (Strüven et al., 2021b). N-3 PUFA, present mainly in fish and fish oil and abundant in the Mediterranean diet, are of particular interest. A higher Mediterranean diet score was associated with higher HRV (Dai et al., 2010), likely mediated by n-3 PUFA intake. These fatty acids may influence HR and HRV directly through autonomic modulation, and indirectly through reductions in inflammatory factors and catecholamines (Drewery et al., 2017). They also appear to regulate cardiac ion channels, improve endothelial function, and reduce inflammatory levels in patients with CVD (Mozaffarian et al., 2008). In one U.S. study, n-3 PUFA intake from any source was associated with greater vagal predominance compared to populations with lower intake (King et al., 2000).

Daily supplementation with n-3 PUFA also shows benefits in secondary prevention. A double-blind randomized controlled trial demonstrated positive effects of fish oil in survivors of MI, possibly through antiarrhythmic effects and increased parasympathetic cardiac tone (Christensen et al., 1996). However, perioperative supplementation with n-3 PUFA did not significantly reduce atrial fibrillation compared with placebo (Mozaffarian et al., 2012).

Overall, the reviewed studies indicate that targeted nutritional interventions, particularly those involving n-3 PUFA and B vitamins, may enhance autonomic regulation and improve HRV in patients with CVD. Promising findings include reductions in POAF and shorter hospital stays, especially in perioperative settings. However, evidence on long-term efficacy remains inconsistent, underscoring the need for further high-quality research to establish standardized nutritional protocols in cardiac rehabilitation (Table 2).

**Table 2:** Studies investigating the efficacy of dietary interventions in CVD management and rehabilitation

Author, date	Design	Sample	Population type	Prevention type	Intervention details; timing	Dependent variable	Effects
Dantas et al. (2002)	Pilot study	17	CAD undergoing CABG	Tertiary	Low fat and sugar foods; Hospital discharge to 180 days	BMI, HDL, LDL, Triglycerides, Total cholesterol	= BMI, = HDL, = LDL, = Triglycerides, = Total cholesterol
Grundmann et al. (2018)	Pilot study	76 (CR *n* = 36; Control *n* = 40)	CAD undergoing CABG	Secondary	CR every meal; From preoperative day 7 to preoperative day 1	Serum Creatinine (24 h), AKI, Safe-related events	= Creatinine 24 h, ↓ AKI, = Safe-related events
Shingu et al. (2021)	Randomized controlled trial	30 (L-carnitine *n* = 15; Control *n* = 15)	Aortic valve surgery	Secondary	3 oral doses of 1 g L-carnitine; 9 days perioperatively (pre-op day 2 to post-op day 7)	POAF	↓ POAF
Osterholt et al. (2022)	Randomized controlled trial	115 (LowS *n* = 56; Control *n* = 59)	CAD undergoing CABG	Secondary	LowS diet; From preoperative day 7	AKI (72 h), Serum Creatinine (24–72 h)	= AKI, = Serum Creatinine
Hodis (1995)	Randomized controlled trial	156 (78/78)	CAD undergoing CABG	Tertiary	Vitamin E + diet + drug; Not specified	Vessel diameter	↓ progression of CAD
Mozaffarian et al. (2012)	Randomized controlled trial	1516 (n-3 PUFA 758; Control 758)	CAD undergoing surgery	Tertiary	n-3 PUFA; Loading then 2 g/day until discharge	POAF	↓ POAF
Mozaffarian et al. (2008)	Cohort study	4465	Medicare population	Secondary	Fish & n-3 PUFA diet; Prior year	Vagal activity, baroreceptor, sinoatrial node	↑ vagal activity, ↑ baroreceptor, ↑ sinoatrial node function
Christensen et al. (1996)	Randomized controlled trial	49 (fish oil *n* = 26; control *n* = 23)	Post-MI patients	Tertiary	5.2 g n-3 PUFA daily; After discharge for 12 weeks	SDNN	↑ SDNN
King et al. (2000)	Pilot study	30 (Groups A, B, C)	CAD undergoing CABG	Tertiary	Diet (CHO/fat ratios); ≥6 weeks post-event	EST, BIA, skinfold thickness	↑ exercise duration, ↑ functional capacity, ↑ HR (Group A)
Feguri et al. (2017)	Randomized controlled trial	57 (CHO + W3, W3, control)	CAD undergoing CABG	Secondary	CHO + intra-op n-3 PUFA; 2 h before anesthesia + during surgery	POAF	↓ POAF
Ogawa et al. (2025)	Randomized controlled trial	44 (CR 22, control 22)	Cardiac surgery	Tertiary	Preoperative; Minimum 14 days before surgery	6MWD, grip strength, SPPB, gait speed	↑ 6MWD, ↑ grip strength, ↑ SPPB scores, ↑ gait speed

*CAD* coronary artery disease, *CABG* coronary artery bypass graft, *BMI* body mass index, *HDL* high-density lipoprotein, *LDL* low-density lipoprotein, *CR* calories restriction, *NGAL* neutrophil gelatinase–associated lipocalin, *AKI* acute kidney injury, *POAF* postoperative atrial fibrillation, *LowS* low serum, *SAA* sulfur-containing amino acids, *n-3 PUFA* omega-3 polyunsaturated fatty acids, *CHO* carbohydrate, *SDNN* standard deviation of normal-to-normal interval, *EST* electrocardiogram stress test, *BIA* bioelectrical impedance analysis, *6MWD* 6-min walking distance, *SPPB* Short Physical Performance Battery, ↓ significant decrease,↑ significant increase, = no significant variation

## Discussion

The purpose of this review was to examine the effectiveness of HRVBF and cardioprotective dietary interventions as secondary and tertiary rehabilitation strategies for managing CVD in the immediate postoperative period. The key findings suggest that, while both approaches have been widely studied in long-term recovery, they have received limited attention in the acute postoperative phase, where they could significantly influence autonomic regulation, inflammation control, and hospital stay duration. Despite this, promising potential emerged for the combined use of HRVBF and specific nutrition to optimize recovery and reduce complication risks during postoperative rehabilitation.

The results of this review align with previous studies suggesting significant benefits of HRVBF and dietary modifications for cardiovascular health, particularly with regard to optimizing autonomic balance and managing inflammation (Lehrer et al., 2013; Shivappa et al., 2018). However, the existing literature has primarily focused on long-term effects, while our findings highlight the lack of studies applying these strategies in the immediate postoperative phase. This gap suggests that integrating HRVBF and cardioprotective nutrition during early rehabilitation could offer a new avenue for improving recovery and minimizing complications, an area that remains underexplored.

Among the various methods aimed at improving cardiovascular health and function, this review focused on HRVBF and dietary interventions to systematically assess the evidence supporting their rationale and potential advantages as secondary and tertiary strategies to prevent relapse and manage CVD. While reviewing the literature on this topic, it became evident that compared with the extensive research on risk factors, prevention, and interventions, there is little evidence on the use of these two approaches after cardiac surgery. Furthermore, few studies have examined their application in the immediate postoperative phase, with limited attention given to indicators of efficacy during this stage, such as hospital stay duration. This contrasts with the more extensively studied long-term rehabilitation effects practices and underscores the need for further investigation.

Promising beneficial effects of HRVBF and cardioprotective dietary interventions emerged that could be particularly valuable in the rehabilitation phase. Examples include optimizing autonomic balance, regulating blood pressure, and modulating inflammation and oxidative stress. Regarding autonomic activity, both practices positively influence the balance between the sympathetic and parasympathetic nervous systems, favoring parasympathetic dominance. These effects could be especially advantageous in the rehabilitative phase, when restoring optimal cardiac regulation as quickly as possible is critical.

Another pathway through which these interventions appear to be beneficial is their anti-inflammatory action (Lehrer et al., 2013; Shivappa et al., 2018). In the immediate postoperative phase after cardiovascular surgery, widespread inflammation is common, largely due to extracorporeal circulation and immune responses aimed at repairing tissue damaged. Although these mechanisms are part of normal healing, excessive or prolonged inflammation can cause serious complications (Jakob, 2001). Controlling and managing postoperative inflammation through HRVBF and an anti-inflammatory diet could therefore be crucial for promoting recovery.

As emphasized, most existing research has focused on the long-term applications. In contrast, effective rehabilitation methods in the immediate postoperative period after cardiac surgery have received little attention. Successful rehabilitation strategies in the early days after cardiac surgery are not well documented, particularly in the hospital setting. A key advantage of HRVBF and nutritional interventions is their feasibility for implementation as early as the initial postoperative period, with the potential to enhance recovery and improve cardiac function.

Both approaches are associated with better autonomic regulation. While HRVBF directly increases HRV, dietary measures may act more indirectly. Foods and dietary components typical of the Mediterranean diet—including n-3 PUFA, B vitamins, probiotics, polyphenols, and weight management—have been linked to both acute and long-term improvements in HRV. These benefits in autonomic function may reduce systolic blood pressure (Nolan et al., 2005), decrease inflammation, and improve cardiovascular prognosis (Burlacu et al., 2021; Fournié et al., 2021). Collectively, these beneficial effects could enhance recovery, reduce hospital stay duration, lower infection risks, and improve the cost-effectiveness of hospitalization.

Nevertheless, some drawbacks should be considered, particularly for HRVBF in the hospital setting. Deep breathing techniques could compromise sternal osteosynthesis and delay wound healing in patients at higher risk of sternal dehiscence (Alhalawani & Towler, 2013). Moreover, for rehabilitation to be effective, training must begin promptly, creating a delicate balance between benefits and risks. Complications such as infections or diastasis should be mitigated through careful assessment of frailty and comorbidities, particularly in elderly patients, and by close daily monitoring during training—factors that may limit the feasibility of HRVBF for all patients.

Dietary strategies require similar caution and control before and after cardiac surgery (Lopez-Delgado et al., 2019). Malnutrition, especially sarcopenia in elderly patients, is associated with worse postoperative outcomes and longer intensive care unit stays. Malnutrition can also exacerbate the inflammatory response to surgical trauma and cardiopulmonary bypass. Pre-existing conditions, such as chronic heart failure, may further impair nutrient absorption due to bowel wall edema. Nutritional support (oral, enteral, or parenteral) after surgery helps maintain gut integrity and modulate oxidative stress and inflammation (Lopez-Delgado et al., 2019). Early nutrition is recommended by international societies for hemodynamically stable patients (Hill et al., 2018). However, to prevent gastrointestinal complications and optimize recovery, nutritional support requires close monitoring. In addition to selecting cardioprotective nutrients, the timing of reintroducing solid foods should be carefully evaluated through a multidisciplinary approach.

Beyond limitations specific to individual treatments, this review may be subject to broader issues. One primary limitation lies in publication bias. Publication bias represents a primary concern: while some studies report non-significant results (Adams et al., 2009), authors and journals tend to publish only significant findings, potentially skewing the literature. Although cross-sectional studies are not interventional, they were initially considered for inclusion to identify mechanisms and associations that could support future clinical trials. Given the limited number of studies on this topic, including diverse designs at the screening stage was necessary to maximize comprehensiveness and generate hypotheses for future research.

Another limitation is the heterogeneity among the included studies regarding methodologies, variables, and outcomes, particularly in nutrition research. This complicates direct comparisons and affects generalizability. A further limitation is the predominant focus on short-term outcomes, with little evaluation of long-term clinical endpoints such as readmission or mortality. This restricts conclusions about the sustained impact of HRVBF and nutritional interventions. Future research should therefore include extended follow-up and hard clinical outcomes. Although the number of studies retrieved was modest, this reflects the specificity of our inclusion criteria, which focused on post–cardiac surgery patients and secondary/tertiary rehabilitation. The search was updated in May 2025 and supplemented by manual reference screening and citation tracking, confirming that despite comprehensive efforts, the eligible literature remains limited.

Finally, restricting inclusion to English-language studies may introduce selection bias, excluding valuable evidence published in other languages and potentially limiting the global relevance of the findings.

## Conclusions and Future Directions

Given the benefits of the two rehabilitation approaches discussed, their compatibility, and the potential role of HRV modulation in mediating nutritional effects on cardiovascular health, an integrated protocol involving both HRVBF and dietary intervention in the immediate postoperative period appears ideal.

Currently, no specific guidelines exist for very early rehabilitation. However, the literature reviewed here—combining knowledge of cardiovascular risk, nonsurgical CVD management, and limited rehabilitation insights—helps identify strategies that could form the foundation of such protocols.

Unlike protocols in previous studies, HRVBF sessions and a specific nutritional plan could be introduced when patients are transferred from the intensive care unit to the ward (typically the third postoperative day) and continue throughout hospitalization. Evidence-based HRVBF protocols recommend five sessions with a facilitator plus daily home practice (2 × 20 min), which is sufficient to produce improvements across physical and psychological outcomes (Lehrer et al., 2013). Considering asthenia, dyspnea, immobility, and other postoperative challenges, shorter sessions may be more feasible (Limmer et al., 2022). After discharge, patients may continue diaphragmatic breathing until follow-up visits, where progress can be assessed.

Regarding diet, evidence on specific postoperative protocols is limited. Nevertheless, strong data support the benefits of the Mediterranean diet, which reduces blood pressure, arterial stiffness, and lipid levels. Thus, it represents a solid foundation for a tailored postoperative diet. Key nutrients such as n-3 PUFA, peptides, and branched-chain amino acids further improve endothelial function, vasodilation, atherosclerosis progression, blood pressure, and postoperative atrial fibrillation, and shorten hospital stays (Colussi et al., 2017). A comprehensive dietary model should also address the timing of solid food reintroduction, food preparation methods, and processing levels. Importantly, structured diets should replace simple dietary advice. This requires the involvement of a nutrition specialist and a team of dietitians to guide patients through tailored plans and periodic assessments, ensuring better compliance and more consistent results.

Similar benefits apply to biofeedback, where specialized staff can provide personalized guidance, adapt protocols, and monitor progress, ensuring effective implementation.

This review highlights the potential advantages of an integrated protocol combining HRVBF and cardioprotective nutrition, particularly in the immediate postoperative period. The hospital setting offers an optimal environment for introducing such programs, allowing standardized implementation and rigorous monitoring by trained staff.

## Data Availability

No datasets were generated or analysed during the current study.
